# High expression of *RABL6* promotes cell proliferation and predicts poor prognosis in esophageal squamous cell carcinoma

**DOI:** 10.1186/s12885-020-07068-w

**Published:** 2020-06-29

**Authors:** Yanfen Feng, Shumei Yan, Yuhua Huang, Qitao Huang, Fang Wang, Yiyan Lei

**Affiliations:** 1 State Key Laboratory of Oncology in South China, Collaborative Innovation Center for Cancer Medicine, Sun Yat-sen University Cancer Center, Guangzhou, 510060 Guangdong China; 2 Department of Pathology, Sun Yat-sen University Cancer Center, Guangzhou, 510060 Guangdong China; 3 Department of Molecular Diagnostics, Sun Yat-sen University Cancer Center, Guangzhou, 510060 China; 4 Department of Thoracic Surgery, the First Affiliated Hospital, Sun Yat-sen University, Guangzhou, Guangdong 510080 People’s Republic of China

**Keywords:** Esophageal squamous cell carcinoma, *RABL6*, Proliferation, Prognosis

## Abstract

**Background:**

Esophageal squamous cell carcinoma (ESCC) is a common malignant carcinoma of digestive system with high mortality. RAB, member RAS oncogene family like 6 (*RABL6*), a member of the RAS subfamily, has been reported as an important molecule in several cancers. However, its potential role in ESCC still remains unclear.

**Methods:**

*RABL6* mRNA expression was detected in 93 frozen ESCC samples using quantitative reverse transcription-polymerase chain reaction (qRT-PCR). Immunohistochemistry was applied to evaluate the RABL6 expression in tissue microarray containing 171 pairs of ESCC tissues and paired para-cancerous tissues. We evaluated RABL6 expression and its correlation with clinicopathological characteristics and survival. Subsequently, the impact of *RABL6* knockdown on the ability of cell proliferation, apoptosis, migration and epithelial-mesenchymal transition (EMT) of ESCC cells was investigated by MTS, Focus formation, flow cytometry, Transwell assays, qRT-PCR, western blot, inverted microscope observation and phalloidin staining, respectively.

**Results:**

Compared to paired para-cancerous tissues, RABL6 was highly expressed in ESCC. The RABL6 high-expression was associated with worse prognosis. We also revealed silencing of *RABL6* caused inhibition of cell proliferation, invasion and migration. Further experiments demonstrated that knockdown of *RABL6* suppressed the aggressive biological activities of ESCC by suppressing EMT in ESCC cells.

**Conclusions:**

*RABL6* functions as a tumor oncogene in ESCC. It would be a potential biomarker predicting prognosis, and a novelty target for ESCC therapy.

## Background

Esophageal cancer (EC) is one of the most common malignant carcinomas worldwide. It ranks the seventh in morbidity and sixth in mortality respectively overall global [1]. It is estimated that 4,779,000 new cases were diagnosed in China in 2015, while 176, 650 new cases in USA in 2019 [2, 3]. EC is also the fifth leading causes of deaths due to cancers in China [2]. For histological subtype, over 90% of EC cases are squamous cell carcinoma (ESCC). Although advances have been made in multiple therapeutic approaches, including surgery, radiotherapy, chemotherapy and combination therapy, the prognosis is still dissatisfactory. Currently, the exact oncogenic molecular mechanisms of ESCC remain unclarified. And effective prognostic biomarkers haven’t been found yet. Herein, discovering new prognostic predicting marker is important, especially in personalized treatment era.

*RABL6* is a novel gene, also known as chromosome 9 open reading frame 86 (C9orf86), or Rab-like protein 1 (RBEL1), or partner of alternative reading frame protein (PARF). Data from the National Center for Biotechnology Information (NCBI) shows that it located at 9q34.3 [4]. It is reported that *RABL6* is a member of the Ras subfamily which are made up of small GTPases. The GTPases have important effect in various cellular functions, such as cell proliferation, differentiation, survival, and so on [5]. Recently, non-small cell lung cancer (NSCLC), breast cancer and pancreatic ductal adenocarcinoma have been showed to overexpress RABL6, and that was closely correlated with poor prognosis [6–9]. In breast cancer cell lines SK-BR-3 and MCF-7, silencing of *RABL6* by siRNA suppressed cell proliferation and invasion capabilities in vitro [7]. Similarly, knocking down of *RABL6* in osteosarcoma cells also impaired cell colony formation and proliferation [10]. These data implicated that *RABL6* is probably a potential oncogene and therapeutic target in cancers.

However, the role of *RABL6* in ESCC has not been studied so far. Herein, we carried out this study, to explore the correlation of RABL6 expression and the clinicopathological characteristic as well as prognosis. Furthermore, the function of RABL6 in the tumorigenesis of ESCC was studied by silencing of *RABL6* in vitro.

## Methods

### Patients and sample collection

In this study, 171 ESCC cases, who received treatment of esophagectomy in the department of thoracic surgery, Sun Yat-sen university cancer center (SYSUCC) (Guangzhou, China) from November 2000 to November 2007, were enrolled. Histological diagnosis was confirmed by pathologists. Patients who have accepted preoperative chemotherapy or radiation, or had other malignant tumors were excluded. We collected patients’ information about their clinical data and pathological characteristics from patients’ medical records. We defined Overall survival (OS) as the date on which patients underwent surgery to the date on which patients been last followed, or death due to any cause. The ethics committee of SYSUCC approved this study.

### Immunohistochemistry (IHC)

IHC staining was performed with a tissue microarray which contained two cancer tissues and one matched esophageal normal tissue of 171 cases of ESCC, to measure the expression of RABL6. Primary antibody was by use of a mouse monoclonal antibody against RABL6 (No.400055684-A01, with a dilution of 1:1200, Abnova). Positive control was using a slide with known immuno-reactivity with RABL6, while negative control was using normal rabbit serum. Two pathologists reviewed the IHC slides, counted positive tumor cell percentage in five representative fields to evaluate the expression of RABL6 expression and determine the IHC scores independently. The patients’ clinical characteristics was not displayed to the pathologists. Staining extent was scored basing on the proportion of cells with immuno-reactivity in a microscope field: 0, 0–10% of cells stained; 1, 10–25% of cells stained; 2, 26–50% of cells stained; 3, 51–75% of cells stained; 4, 76–100% of cells stained. And staining intensity varying from weak to strong marked as different scores: negative marked 0, light yellow marked 1, heavy yellow marked 2, and brown marked 3. Any disagreement of immunohistochemical result analysis was discussed and achieved consensus by these two pathologists. If consensus was still not achieved, a third senior pathologist made the decision. The overall score was the result of staining extent score multiplied by staining intensity score. High expression was defined as scores higher than or equal to median score, and low expression was defined as scores less than median score.

### Cell culture and transfection

The NE1 immortalized esophageal epithelial cell line was obtained from Professor Libing Song from SYSUCC, while the Het-1A cell line was applied by the American Type Culture Collection (ATCC, Manassas, VA, United States). ESCC cell line EC9706 were obtained from TOKU-E Company (Bellingham, WA, United States); Leibniz-Institut DSMZ (Braunschweig, Germany) supplied cell lines KYSE30, KYSE150, KYSE180 and KYSE510; TE-1, TE-9, TE-2, TE-11 and TE-5 were purchased from the RIKEN BRC Cell Bank; Deutsche Sammlung von Mikroorganismen und Zellkulturen (DSMZ, Braunschweig, Germany) supplied cell lines KYSE520 and HK1. The NE1 and Het-1A cells were stored in mixed serum-free medium and EpiLife medium (Invitrogen, Carlsbad, CA, United States); while all cell lines were authenticated before used according to STR fingerprinting as described previously [11]. *RABL6* knockdown was accomplished by use of small interfering RNA (siRNA). RNA interference siRNA oligonucleotides and non-targeting siRNA were purchased from OBiO Company (Shanghai, China). We seeded ESCC cells in dishes with 6 wells. And 2 × 10^5^ cells were seeded per well. Twenty-four hours after seeding, knockdown experiments were done. Cells were transfected by using Lipofectamine 2000, supplied by Invitrogen (Carlsbad, CA, United States). Following the instructions of manufacturer, we transfected cells with 50 nanomoles negative control siRNA (NC) or two *RABL6* siRNA (*RABL6*-siRNA) duplex oligonucleotides, *RABL6*-siRNA#1: GGCCTAAAGTACCTTCATA; and *RABL6*-siRNA#2: GTCATGATGTTCGACATTA. Based on the WB results of RABL6 expression by ESCC cell lines, TE-2 and YES-2 were not the highest expression cell lines, however we observed the most significant morphology changes after *RABL6* knockdown in these two cell lines among all cell lines we tested. So, we chose YES2 and TE2 cell lines to perform downstream experiments.

### Cell proliferation

MTS assay (Promega, Madison, WI, United States) was used to evaluate cell proliferation. We carried out the assay according to the experimental protocol described previously [11]. Briefly, 1500 cells were seeded into a plate with 96 wells plates with 200 μL media, and cultured to the specified days. Then 20 μL MTS solution was added in the plates incubated for another 2 h. Finally, by use of an enzymatic-reader (Thermo Scientific, Waltham, MA, United States), each well’s optical density was measured at 490 nm. We repeated independent experiments for 3 times. The data were expressed as Mean ± Standard Error of Mean (SEM).

### Focus formation

Focus formation was performed as described previously [11]. Briefly, in a plate with 6 wells, we plated 500 cells inside. After 10 days, we fixed surviving colonies, stained them by using crystal violet staining and counted cell colonies. Independent experiments were done triple times, and the data were showed in the form of Mean ± SEM.

### Transwell migration and invasion assays

Transwell chambers inserts for 24-well plates were supplied by Corning Incorporated (New York, United States). Briefly, in the upper chamber, 1 × 10^5^ cells/ well in 200 μL medium were seeded. And in the lower chamber, we added 600 μL of medium (with 80% FBS) to conduct a chemoattractant. Cells were incubated for 24 h at 37 °C. And then a cotton swab was used to remove the remaining cells on the surface of the upper. Cells migrated to the bottom of the filters were counted under microscope. Before counting, the cells were fixed by 4% formaldehyde and stained with 0.5% crystal violet. And then we counted the cells in five photographed fields.

### Apoptotic analysis

Annexin V-FITC/PI Apoptosis Detection Kit (BD Biosciences, Franklin Lakes, United States) was used to detect apoptotic cells according to instruction of manufacturer. After transfection, the cells were treated by with Annixin V and PI, and examined by the Flow cytometry (BD Biosciences, San Diego, United States).

### RNA extraction and qRT-PCR

The methods of RNA extraction and qRT-PCR were conducted as what was reported previously in the literature [11]. We extracted total RNA and conducted reverse transcription by using Trizol Reagent and Superscript III Reverse Transcriptase (Invitrogen, Carlsbad, CA, United States) respectively. We listed the primer sequences in the Supporting Materials. And the reactions were performed thrice with ABI PRISM 7900 Sequence Detector, with a SYBR Green PCR Kit (Supplied by Biosystems, Carlsbad, CA, United States). The relative expression levels were quantified and analyzed by the SDS 2.3 software (Applied Biosystems, Foster City, CA, United States). GADPH was applied as an endogenous reference.

### Western blot analysis

The protocol of western blot assay were performed as reported previous [11]. The dilution of RABL6 (No.400055684-A01), E-cadherin (ab15148), β-catenin (ab16051), Vimentin (ab8978) and slug (ab106077) used for western blot was 1:1000, while the dilution of a-tubulin and GADPH was 1:5000. β–catenin, slug, a-tubulin (ab7291), GADPH (ab181602), E-cadherin and Vimentin was applied by Cell Signaling Technology (Beverly, MA, United States). GADPH and a-tubulin was probed on the membranes as an internal control antibody, for the sake of confirming equal loading.

### Morphological observation and phalloidin staining

Cell morphology changes of ESCC with or without knocking down of *RABL6* were observed by inverted microscope, and representative images were taken. For phalloidin staining, cells growing on the glass slide were fixed in 4% formaldehyde at room temperature for half an hour, and then we rinsed the slides 3 times with PBS. After that, phalloidin-conjugate working solution (Phalloidin-iFluor 555 Reagent, ab176756) was added on the slides and incubated at room temperature for an hour. And we rinsed all slides 3 times with PBS. DAPI (blue) was applied to stain nuclear DNA. Finally, we observed cell morphology change under the fluorescence microscope, and took representative images.

### Statistical analysis

The data were recorded as Mean ± SEM and analyzed through Graphpad prism software (San Diego, CA, United States). One-way analysis of variance (ANOVA) and Newman Keul’s multiple comparison tests were applied to analyze the significant differences of more than two groups. The expression of RABL6 between tumor samples and matched non-cancerous samples was analyzed by paired t-test. Survival data were analyzed through Kaplan–Meier survival curves. Independent prognostic factors were indentified by cox proportional hazards regression model. *P* value < 0.05 was set as statistically significant.

## Results

### RABL6 was overexpressed by ESCC tissues and predicted worse prognosis

**Fig. 1:**
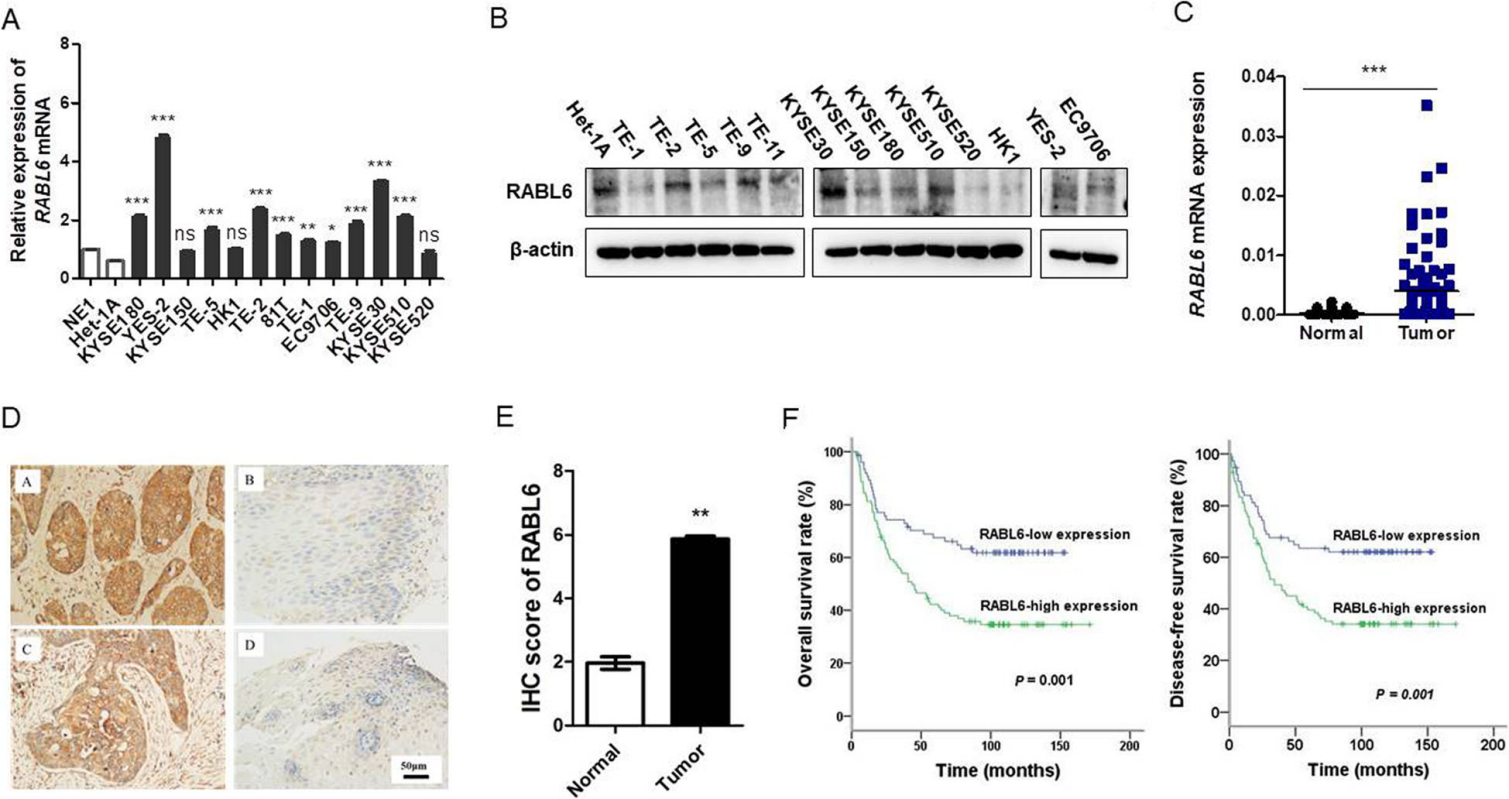
*RABL6* is highly-expressed by ESCC and is associated with worse prognosis. **a***RABL6* expression was tested and compared between normal esophageal epithelial cell line NE-1 and Het-1 and all tested ESCC cell lines via qRT-PCR. **b** Expression of *RABL6* in different ESCC cell lines and in normal esophageal epithelial cell line Het-1A was compared by Western blot analysis; the original full-length gels are presented in Supplementary Figure S1. **c-d** The scores of RABL6 expression in 171 ESCC cancerous samples were compared with matched non-cancerous samples by immunohistochemistry staining. **c** Representative images of *RABL6* IHC staining in 2 pairs of ESCC cases (original magnification: 100x). **d** Results are expressed in the form of mean+/−SEM. **e- f** Kaplan-Meier analysis showed the overall survival rate and disease-free survival rate of ESCC patients stratified by *RABL6* expression. * indicates *P* < 0.05, ** indicates *P* < 0.01, *** indicates *P* < 0.01 for statistical results

The expression levels of *RABL6*were compared between normal esophageal epithelial cell line NE-1 and Het-1A and all tested cancer cell lines by qRT-PCR. The average fold change of *RABL6* mRNA was obviously higher in all tested cancer cell lines compared with NE-1 and Het-1A, except KYSE150 (Fig. 1a). Compared with Het-1A, western blot analysis showed that RABL6 expression was higher in cancer cell lines (Fig. 1b). To compare the expression of RABL6, two independent sets of human samples were used. Fresh tissues of 93 ESCC cases and paired non-cancerous tissues were tested by qRT-PCR; while an ESCC tissue microarray (including 171 informative pairs of cancerous and corresponding non-cancerous tissues) were tested by IHC staining with a monoclonal RABL6 antibody, respectively. QRT-PCR results demonstrated that in cancerous tissues, the average fold change of *RABL6* mRNA was obviously higher than those in paired non-cancerous and prognosis of ESCC patients was analyzed statistically. For the IHC staining results, the median score was set at 5. According to the final scores, we divided all cases into two groups: the high RABL6 expression group, with the scores ≥5, and the low RABL6 expression group, with the scores < 5. The median overall survival rate (OS) and median disease-free survival rate (DFS) of all cases with RABL6 high-expression was significantly lower than those with low-expression (44.4 months vs. 70.4 months, *P* = 0.001 and 39.7 months vs. 94.0 months, *P* = 0.001, respectively) by Kaplan-Meier analysis. Multivariate analysis indicated that RABL6 was an independent prognostic factor in this cohort of 171 resected ESCC patients (HR = 1.631; 95% CI, 1.03–2.59; *P* = 0.038, Table 1).

**Table 1:** Univariate and multivariate Cox Regression analyzes for overall survival (OS) in ESCC patients

Factors	HR (95% CI)	*P* value	HR (95% CI)	*P* value
Age (≤ 55 vs. > 55)	0.956 (0.63–1.45)	0.833	–	–
Gender	0.468 (0.27–0.83)	**0.009**	0.608 (0.34–1.08)	0.092
Tumor location	0.813 (0.55–1.19)	0.293	–	–
Lymph-vascular invasion	0.479 (0.31–0.73)	**0.001**	0.479 (0.31–0.73)	**0.001**
Perineural invasion	0.652 (0.43–0.99)	**0.044**	0.766 (0.49–1.19)	0.243
Histological differentiation	1.666 (1.22–2.27)	**0.001**	1.396 (1.02–1.90)	**0.035**
Pathological Stage	3.458 (2.26–5.29)	**0.000**	2.233 (1.09–4.58)	**0.028**
Lymph node metastasis	3.650 (2.33–5.71)	**0.000**	1.566 (0.74–3.31)	0.240
RABL6 expression (High vs. low)	2.119 (1.35–3.32)	**0.001**	1.631 (1.03–2.59)	**0.038**

*HR* Hazard ratio, *CI* confidential interval

### RABL6 expression was obviously correlated with lymph-vascular invasion

Clinical parameters of the 171 ESCC patients, including age, gender, tumor location, lymph-vascular invasion, peri-neural invasion, histological differentiation, pathological stage and lymph node metastatic status are summarized in Table 2. The location of tumor was defined as upper esophagus, middle esophagus, and lower esophagus, according to where the tumors located. Pathological stage was defined according to the criteria of the AJCC (2017 version). Patients’ median age in this study was 55.0 years (range: 30.0–75.0 years). There were 129 (75.5%) males and 42 (24.6%) females (male to female ratio, 3.07:1). RABL6 expression was associated with patients’ age and lymph-vascular invasion (*P* = 0.040 and *P* < 0.000, respectively). High expression of RABL6 was more common in patients older than 55 years, and in patients with lymph-vascular invasion. No statistical association was found between expression of RABL6 and other clinicopathological characteristics such as gender, tumor location, peri-neural invasion, histological differentiation, pathological stage, and lymph node metastatic status in our study (Table 2).

**Table 2:** Baseline characteristics of ESCC patients and the correlation with RABL6 expression

Characteristics	Total [cases (%)]	RABL6 expression [cases (%)]		*P* value
	*N* = 171	High *N* = 96	Low *N* = 75	
**Age (years)**				**0.040**
≤ 55	79 (46.2)	45 (26.3)	34 (19.9)	
> 55	92 (53.8)	51 (29.8)	41 (24.0)	
**Gender**				0.320
Male	129 (75.4)	74 (43.2)	55 (32.2)	
Female	42 (24.6)	22 (12.9)	20 (11.7)	
**Tumor location**				0.132
Upper	13 (7.6)	8 (4.7)	5 (2.9)	
Middle	114 (66.7)	69 (40.4)	45 (26.3)	
Low	44 (25.7)	19 (11.1)	25 (14.6)	
**Lymph-vascular invasion**				**0.000**
Yes	79 (46.2)	56 (32.7)	23 (13.5)	
No	92 (53.8)	40 (23.4)	52 (30.4)	
**Perineural invasion**				0.139
Yes	63 (36.8)	40 (23.4)	23 (13.5)	
No	108 (63.2)	56 (32.7)	52 (30.4)	
**Histological differentiation**				0.282
Well	39 (22.8)	22 (12.9)	17 (9.9)	
Moderate	93 (54.4)	48 (28.1)	45 (26.3)	
Poor	39 (22.8)	26 (15.2)	13 (7.6)	
**Pathological stage**				0.374
I & II	103 (60.2)	55 (32.2)	48 (28.1)	
III & IV	68 (39.8)	41 (23.9)	27 (15.8)	
**Lymph node metastasis**				0.162
No	90 (52.6)	46 (26.9)	44 (25.7)	
Yes	81 (47.4)	50 (56.1)	31 (18.1)	

### Knockdown of *RABL6* inhibited proliferation of cancer cells

**Fig. 2:**
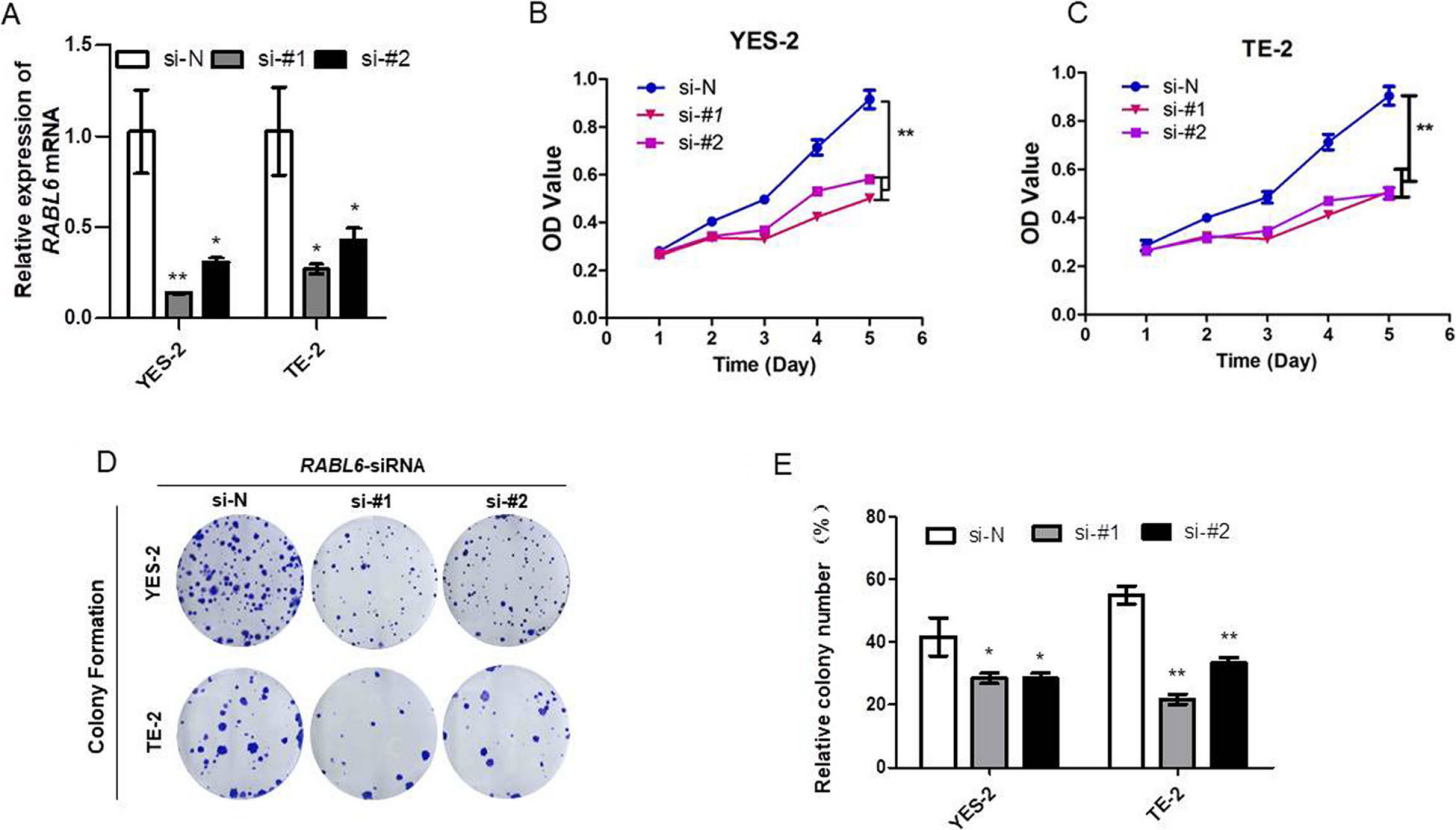
Knockdown of *RABL6* suppressed ESCC cell proliferation. **a-e** YES-2 and TE-2 cells were transfected with siRNAs specifically targeting *RABL6* (si-#1, si-#2) or scrambled siRNA control (si-N). **a** QRT-PCR confirmed the effective knockdown of *RABL6* in these ESCC cells. **b-c** MTS assay tested the cell growth rate of these *RABL6* knockdown cells, and the results were analyzed statistically. **d-e** Single cell clone assay detected the colony formation. **d** Representative image of clone formation **e** Results are analyzed statistically. The experiments were repeated 3 times independently, and the representative data of the experiments are presented in figures. * indicates *P* < 0.05, ** indicates *P* < 0.01 for statistical results

Owing to RABL6 high-expression predicted poorer prognosis in ESCC patients, we raised a hypothesis that RABL6 might play oncogenic roles in ESCC, and RABL6 expression might promote cancer cell growth. So, the role of RABL6 on cell growth was invested via knockdown of *RABL6*. We used small interfering RNA (siRNA) to knock down *RABL6* in YES-2 and TE-2 cells stably. The expression of *RABL6* in *RABL6*-knocked-down cells was examined via qRT-PCR method (Fig. 2a). MTS assay displayed that cell growth rates in *RABL6*-knocked-down YES-2 and TE-2 cells were obviously lower than the negative control cells (NC) (Fig. 2b, c). The result of focus formation assay showed that the *RABL6*-knocked-down YES-2 and TE-2 cells formed lower number and smaller colonies than the control cells (Fig. 2d, e). All the results indicated that knockdown of *RABL6* inhibited cell proliferation and growth in YES-2 and TE-2 cells.

### Knockdown of *RABL6* had no influence on apoptosis in ESCC cells in vitro

**Fig. 3:**
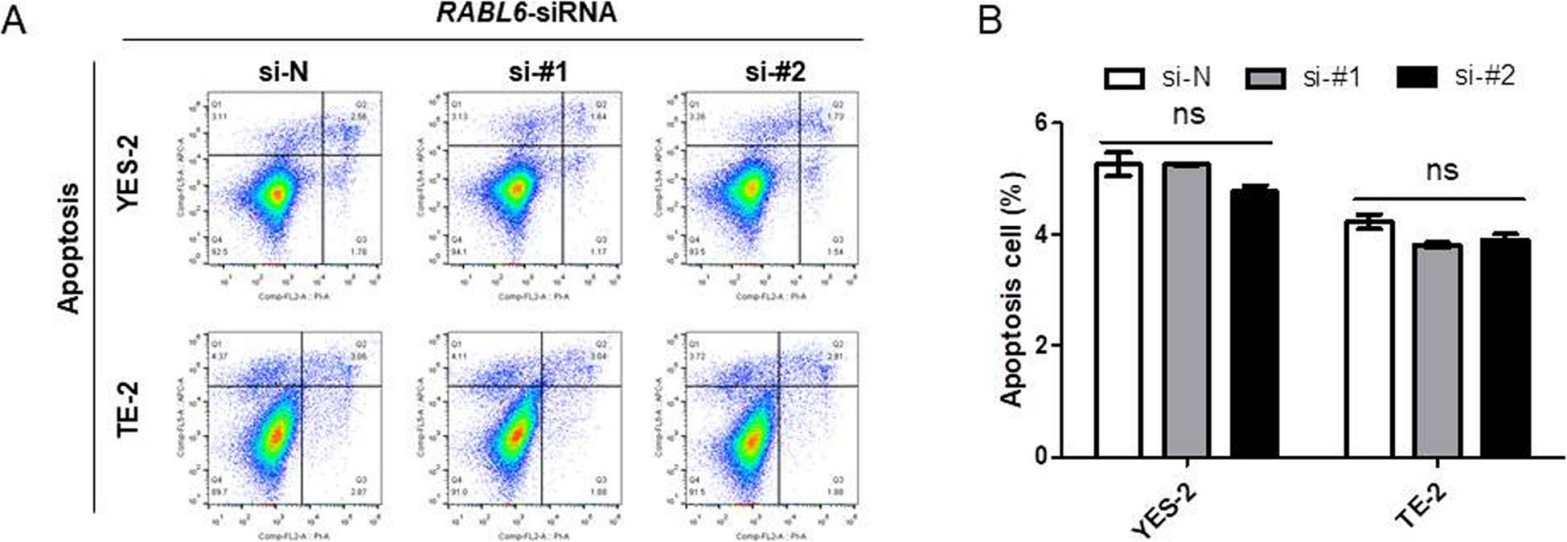
Knockdown of *RABL6* had no impact on ESCC cells. **a** Flow cytometry assay was performed to analyze apoptosis of YES-2 and TE-2 cells with *RABL6* knockdown. **b** Results are statistically analyzed. Values represented the mean ± SD data in triplicate, ns: no significance

The effect of RABL6 in cell apoptosis was evaluated by flow cytometry analysis. Results revealed that *RABL6* silencing couldn’t induce apoptosis in YES-2 and TE1 cells (Fig. 3a, b).

### Knockdown of *RABL6* inhibited migrating and invading of ESCC cells

**Fig. 4:**
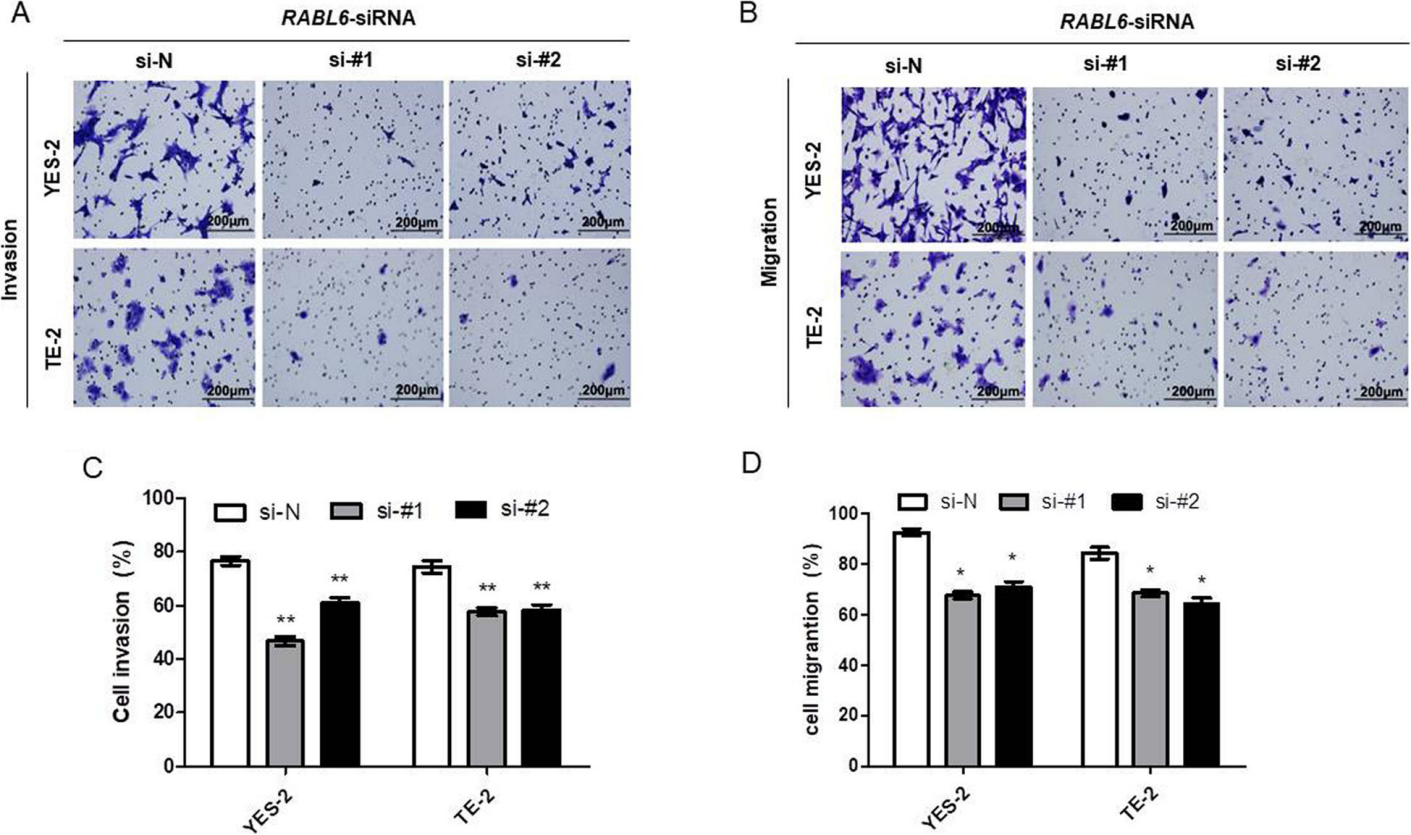
Knockdown of *RABL6* inhibited invading and migrating of ESCC cells. **a-d** Transwell assay was conducted to compare migration and invasion between cells treated with siRNAs (si-#1, si-#2) and si-N. **a-b** Representative images of invasive or migrated cells are provided. **c-d** Results are analyzed statistically. The experiments were repeated 3 times independently, and the representative data of the experiments are presented in figures. * indicates *P* < 0.05, ** indicates *P* < 0.01 for statistical results

Since the statistical results showed that RABL6 expression was closely associated with lymph-vascular invasion, we studied the influence of RABL6 on migration and invasion in ESCC cells through Transwell assay. Results showed that the abilities of migrating and invading of ESCC cells were significantly decreased after silencing of *RABL6* (Fig. 4a, b, c, d). These results indicated that knockdown of *RABL6* remarkably inhibited migrating and invading of cancer cells.

### Knockdown of *RABL6* inhibited epithelial-mesenchymal transition (EMT) in ESCC cells

**Fig. 5:**
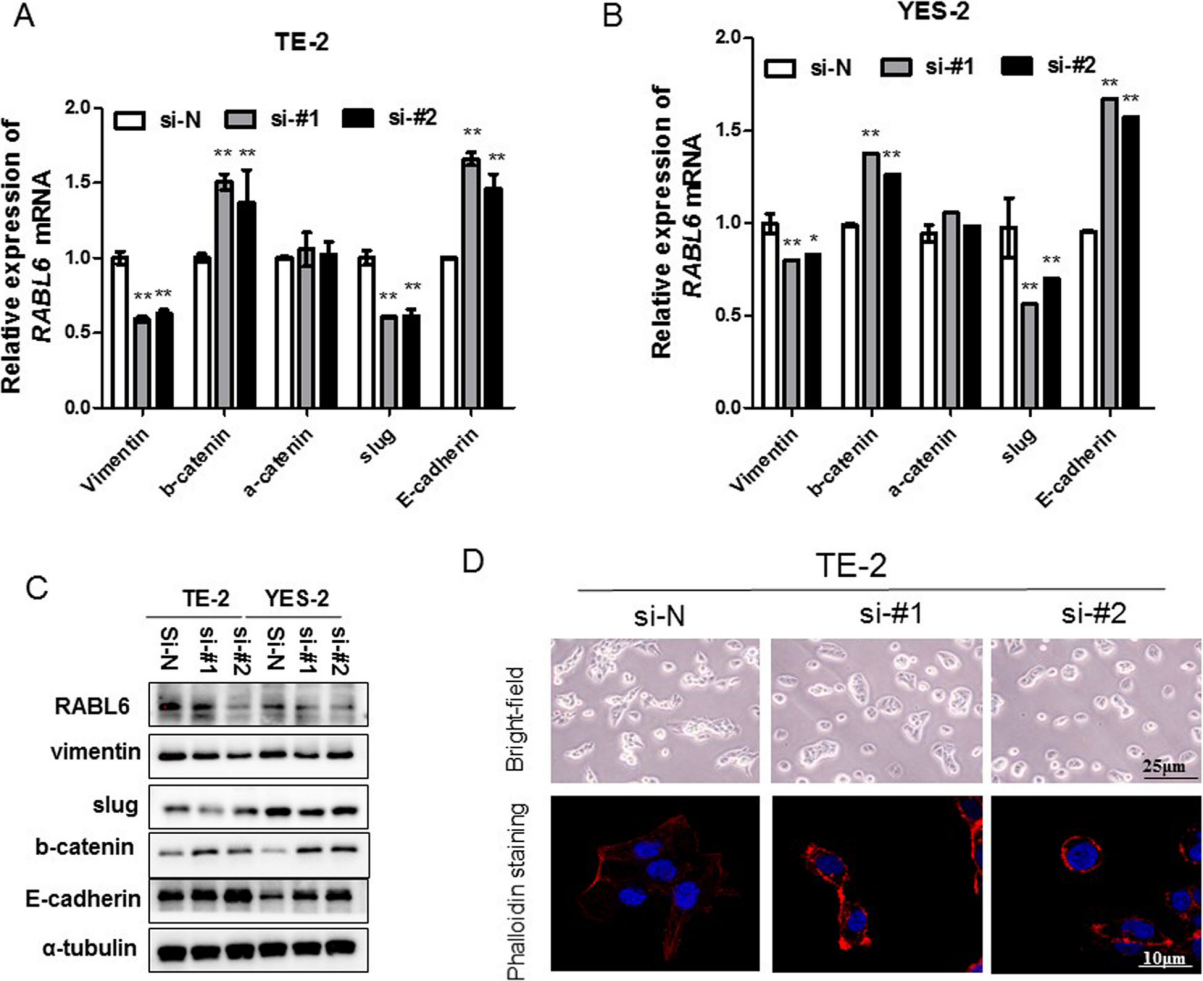
Knockdown of *RABL6* inhibited epithelial-mesenchymal transition in ESCC cells (**a-c**). **a-b** Relative expressions of E-cadherin, a-catenin, b-catenin, Vimentin, and Slug in YES-2 and TE-2 cells were compared by qRT-PCR between siRNA *RABL6*-silenced cells (si#-1, si-#2) and their control cells (si-N). **c** Western blots comparing siRNA *RABL6*-silenced cells (si#-1, si-#2) with their control cells (si-N) are showed with the expression of vimentin, slug, β-catenin and E-cadherin. A-tubulin was taken as control; the original full-length gels are presented in Supplementary Figure S2. **d** Representative bright-field images of ESCC cells and ESCC cells stained with fluorescent phalloidin to show actin structures, with or without silencing of RABL6. DAPI (blue) was applied to stain nuclear DNA. (original magnification of bright-field: 400x; original magnification of fluorescent phalloidin: 1000x). Data are expressed in the form of the mean ± SEM. * indicates *P* < 0.05, ** indicates *P* < 0.01 versus the control

Whether RABL6 promoted tumor cell migration via inducing EMT remains unclear. In order to analyze the function of RABL6 on EMT, we studied the expression of EMT markers and EMT-related transcription factors through qRT-PCR and western blot, and also observed cell morphology changes by inverted microscope in *RABL6* knockdown cells and control cells. QRT-PCR results showed that the expression of epithelial markers E-cadherin and β-catenin was obviously increased, and the mesenchymal marker Vimentin and slug was significantly decreased in knocked-down *RABL6* cells compared to control groups (Fig. 5a, b). The results of western blot were consistent with the results of qRT-PCR (Fig. 5c). We observed by bright field that *RABL6* knockdown ESCC cells were less spindle and became cobble stone like shape compared to their negative control cells in shape. Phalloidin staining showed circumferential actin belts in *RABL6* knockdown ESCC cells, while the actin structures were spindle-shaped in the control cells (Fig. 5d). This indicated the occurrence of EMT. All the results together revealed that silencing of *RABL6* inhibited EMT in ESCC cells.

## Discussion

Esophageal cancer is one of the most common malignant carcinomas worldwide, especially in China. Surgical treatment is a main therapeutic modality for early stage and locally advanced ESCC. For locally advanced cases, neoadjuvant or adjuvant chemotherapies are now applied. For unresectable or late-staged cases, the concurrent chemoradiotherapy maybe the recommended therapy [12]. Nevertheless, the treatment outcome for ESCC is still under satisfactory with these traditional therapeutic methods. Recently, targeted therapy has led to significant breakthroughs in cancer therapy, such as metastatic melanoma, NSCLC, gastrointestinal stromal tumor, and so on [13]. For ESCC, some studies reveal that mTOR, PTEN, and Forkhead box M1 (FOXM1) maybe the prognostic predictors and therapeutic targets [14, 15]. However, effective targeted therapy for ESCC is still needed to be developed, due to the exact cellular and molecular mechanisms of oncogenesis and progression for ESCC remains unclear.

*RABL6* is a novel Ras superfamily protein. The Ras superfamily of GTPases comprises several subfamilies of small GTP-binding proteins which play pivotal roles in tumorigenesis, as their functions included cell proliferating, differentiating, and apoptosis [16, 17]. Several studies found elevated RABL6 expression in various human cancers, including pancreatic ductal adenocarcinomas, pancreatic neuroendocrine tumors and breast tumors. And RABL6 overexpression was associated with poor survival in those cancers [7–9]. However, its role in ESCC is yet to be discovered. Here we firstly investigated *RABL6* in ESCC. We found that RABL6 was overexpressed in ESCC tissues and cell lines. And patients with high RABL6 expression had statistical poorer prognosis than those with low RABL6 expression. High RABL6 expression was an independent prognostic factor in ESCC. And patients with high expression of RABL6 had obviously higher rate of lymph-vascular invasion compared to those with low expression of RABL6. Studies showed that metastasis and recurrence are key factors contributing to poor prognosis of cancer, and the presence of lymph-vascular invasion strongly associated with high risk of metastasis and recurrence in endometrial cancer, ESCC, and so on [18, 19]. These may be one of the reasons to explain RABL6 high expression associated with poor prognosis, and implied that *RABL6* played a vital role in the malignant progress of ESCC.

To investigate the role of *RABL6*, a series of functional studies were carried out. In vitro studies showed that knockdown of *RABL6* inhibited tumor cell growth, proliferation, invasion and migration. Tang et al. found that silencing of *RABL6* gene in U2-OS and SAOS2 osteosarcoma cell lines suppressed cell colony formation and proliferation in vitro [10]. They also demonstrated that *RABL6* modulated G1-S transition in cell cycle analysis. *RABL6* regulated retinoblastoma 1 (Rb1) activity in osteosarcoma cells, which was the major player in cell cycle control [10, 20, 21]. Li Y et al. also reported that silencing of *RABL6* promoted breast cancer cell growth was via promoting apoptosis [7]. Besides cell cycle and apoptosis, Montalbano et al. reported that *RABL6* knockdown resulted in marked cell growth suppression, which is associated with inhibition of extracellular signal-regulated kinase phosphorylation [22]. These reports indicated that *RABL6* displays an oncogenic function by regulating cell cycle or inducing apoptosis, or influencing signal pathway. However, our study showed that silencing of *RABL6* couldn’t induce apoptosis in ESCC cell in vitro. Whether *RABL6* carried out its oncogenic function in ESCC via regulating cell cycle or signaling pathway needs to be further investigated.

We also demonstrated that knockdown of *RABL6* inhibited cell invasion and migration via EMT. It was reported that EMT inducers were important for morphogenesis and organogenesis through regulating cell migrating. The abnormal activation ofEMT played a key role in development and metastasis in tumors [23]. Up regulation of mesenchymal markers, down regulation of epithelial markers, abnormal localization of β-catenin and nuclear expression of Vimentin are some of its characteristics [24, 25]. In the development of epithelial tumors, cancer cells obtain phenotypes of invasion and motivation through EMT, which results in invasion or metastasis, leading to the death of about 90% of patients [26].

Taken all these together, we found that *RABL6* plays an important part in the tumorigenesis and progression in ESCC.

## Conclusions

Conclusively, we indicated firstly that RABL6 is highly expressed by ESCC and associated with poor prognosis. Its expression is associated with lymph-vascular invasion. Downregulation of *RABL6* suppressed proliferation, migration and EMT of ESCC cells. Thus, *RABL6* exert an important function on the progression of ESCC, and may be promising prognostic marker and a potential therapeutic target for ESCC. Its underlying mechanism and clinical application need to be further developed.

## Supplementary information

**Additional file 1.**

**Additional file 2.**

**Additional file 3.**

## Data Availability

The data in this study are available from the corresponding author on request.

## Abbreviations

*RABL6*: RAB, member RAS oncogene family like 6
C9orf86: Chromosome 9 open reading frame 86
ESCC: Esophageal squamous cell carcinoma
qRT-PCR: Quantitative reverse transcription-polymerase chain reaction
EMT: Epithelial-mesenchymal transition
EC: Esophageal cancer
RBEL1: Rab-like protein 1
PARF: Partner of alternative reading frame protein
NCBI: National Center for Biotechnology Information
NSCLC: Non-small cell lung cancer
OS: Overall survival
ATCC: American Type Culture Collection
siRNA: Small interfering RNA
SDS-PAGE: Sodium dodecyl sulfate-polyacrylamide gel electrophoresis
ANOVA: One-way analysis of variance
NC: Negative control cells
FOXM1: Forkhead box M1
Rb1: Retinoblastoma 1
