# Supplementary figures and images for: High expression of RABL6 promotes cell proliferation and predicts poor prognosis in esophageal squamous cell carcinoma
Source: BMC Cancer. 2020 Jun 29;20:602. doi: 10.1186/s12885-020-07068-w (PMC7325041; doi:10.1186/s12885-020-07068-w)

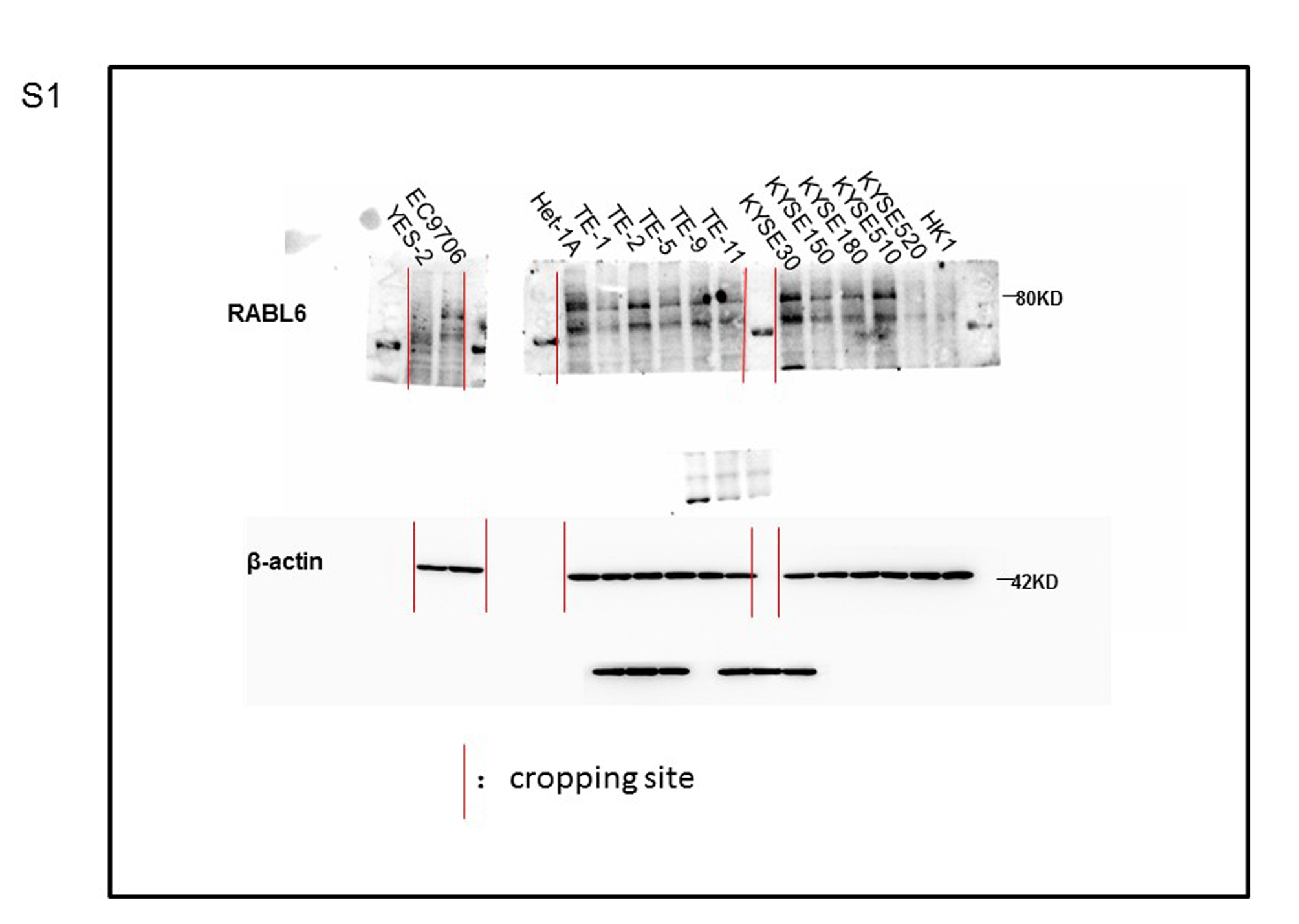

Supplement: Supplementary file 1 — Additional file 1. [file 12885_2020_7068_MOESM1_ESM.tif]

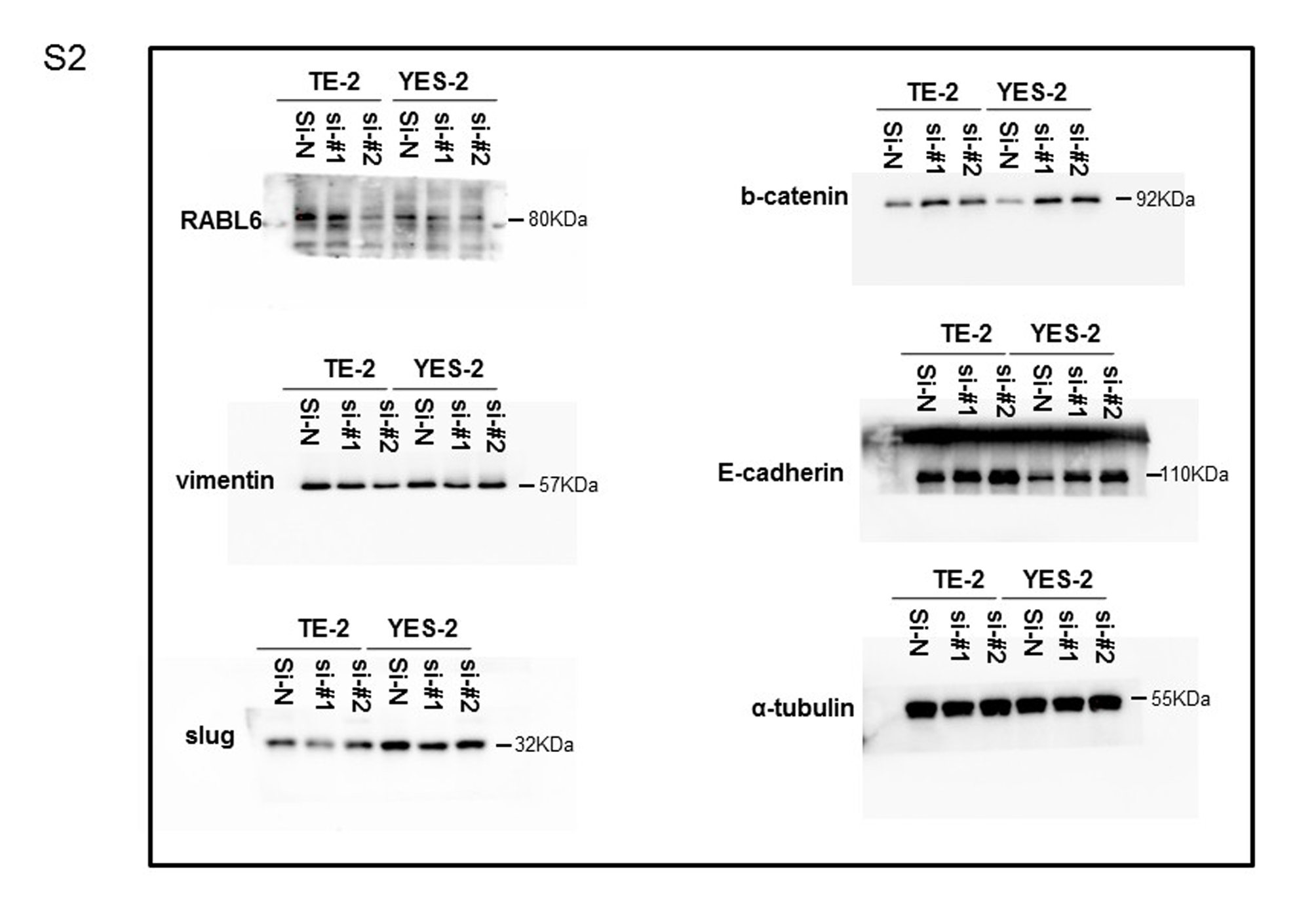

Supplement: Supplementary file 2 — Additional file 2. [file 12885_2020_7068_MOESM2_ESM.tif]
