# Supplementary material for: High expression of RABL6 promotes cell proliferation and predicts poor prognosis in esophageal squamous cell carcinoma
Source: BMC Cancer. 2020 Jun 29;20:602. doi: 10.1186/s12885-020-07068-w (PMC7325041; doi:10.1186/s12885-020-07068-w)
Supplement: Supplementary file 3 — Additional file 3. [file 12885_2020_7068_MOESM3_ESM.docx]

**Supporting materials**

1. **Sequencing of siRNA:**

si#1：GGCCTAAAGTACCTTCATA

si#2：GTCATCATGTTCGACATTA

si-N：CCACGGATGACATCGTGAA

1. **Primer sequencing of markers tested in the study**

*RABL6*

Forward Primer: TGATCCGGGGAGACAGGAAC

Reverse Primer: CGATGTCATCCGTGGTCTTGTA

**E-cadherin**

Forward Primer: TGCCCAGAAAATGAAAAAGG

Reverse Primer: GTGTATGTGGCAATGCGTTC

**a-catenin**

Forward Primer: AGCGAATTGTGGCAGAGTGT

Reverse Primer: GTCTACGCAAGTCCCTGGTC

**β-catenin**

Forward Primer: ACAACTGTTTTGAAAATCCA

Reverse Primer: CGAGTCATTGCATACTGTCC

**Vimentin**

Forward Primer: GAGAACTTTGCCGTTGAAGC

Reverse Primer: GCTTCCTGTAGGTGGCAATC

**Slug**

Forward Primer: GGGGAGAAGCCTTTTTCTTG

Reverse Primer: TCCTCATGTTTGTGCAGGAG

Reverse Primer: GGCCCGTGGAACATAAGTCTT
